# Optical Genome Mapping as a Next-Generation Cytogenomic Tool for Detection of Structural and Copy Number Variations for Prenatal Genomic Analyses

**DOI:** 10.3390/genes12030398

**Published:** 2021-03-11

**Authors:** Nikhil Shri Sahajpal, Hayk Barseghyan, Ravindra Kolhe, Alex Hastie, Alka Chaubey

**Affiliations:** 1 Department of Pathology, Augusta University, Augusta, GA 30912, USA; nsahajpal@augusta.edu (N.S.S.); rkolhe@augusta.edu (R.K.); 2 Center for Genetic Medicine Research, Children’s National Hospital, Washington, DC 20010, USA; haykbarseghyan@ucla.edu; 3 Genomics and Precision Medicine, School of Medicine and Health Sciences, George Washington University, Washington, DC 20037, USA; 4 Bionano Genomics Inc., San Diego, CA 92121, USA; ahastie@bionanogenomics.com

**Keywords:** optical genome mapping, OGM, structural variation, copy number variation, cytogenetics, cytogenomics, prenatal genetic testing, chromosomal aberrations, aneuploidies

## Abstract

Global medical associations (ACOG, ISUOG, ACMG) recommend diagnostic prenatal testing for the detection and prevention of genetic disorders. Historically, cytogenetic methods such as karyotype analysis, fluorescent in situ hybridization (FISH) and chromosomal microarray (CMA) are utilized worldwide to diagnose common syndromes. However, the limitations of each of these methods, either performed in tandem or simultaneously, demonstrates the need of a revolutionary technology that can alleviate the need for multiple technologies. Optical genome mapping (OGM) is a novel method that fills this void by being able to detect all classes of structural variations (SVs), including copy number variations (CNVs). OGM is being adopted by laboratories as a tool for both postnatal constitutional genetic disorders and hematological malignancies. This commentary highlights the potential for OGM to become a standard of care in prenatal genetic testing based on its capability to comprehensively identify large balanced and unbalanced SVs (currently the strength of karyotyping and metaphase FISH), CNVs (by CMA), repeat contraction disorders (by Southern blotting) and multiple repeat expansion disorders (by PCR-based methods or Southern blotting). Next-generation sequencing (NGS) methods are excellent at detecting sequence variants, but they are unable to accurately resolve repeat regions of the genome, which limits their ability to detect all classes of SVs. Notably, multiple molecular methods are used to identify repeat expansion and contraction disorders in routine clinical laboratories around the world. With non-invasive prenatal testing (NIPT) becoming the standard of care screening assay for all global pregnancies, we anticipate that OGM can provide a high-resolution, cytogenomic assay to be employed following a positive NIPT screen or for high-risk pregnancies with an abnormal ultrasound. Accurate detection of all types of genetic disorders by OGM, such as liveborn aneuploidies, sex chromosome anomalies, microdeletion/microduplication syndromes, repeat expansion/contraction disorders is key to reducing the global burden of genetic disorders.

## 1. Introduction

Global medical associations, such as the American College of Obstetrics and Gynecology (ACOG), the International Society of Ultrasound and Obstetrics and Gynecology (ISUOG), and the American College of Medical Genetics and Genomics (ACMG), recommend prenatal genetic testing that includes screening with non-invasive prenatal testing (NIPT) and invasive diagnostic testing be offered to all pregnant women, irrespective of the gestation age and maternal age [1,2,3]. Typically, NIPT can be offered to pregnant women as early as ≤10 weeks of gestation [4]. Following a positive screen or a “no-call” result that may be due to technical limitations of each NIPT platform, invasive diagnostic testing is recommended to confirm the findings of the screening test. Additionally, pregnancies that have an abnormal ultrasound showing fetal defects are recommended to be followed up with diagnostic invasive testing, which includes chorionic villus sampling (CVS), amniocentesis or periumbilical blood sampling (PUBS) performed at either 10–14 weeks or >15 weeks of pregnancy [5].

Since the early 1980s, confirmatory diagnostic testing included conventional cytogenetic methods such as karyotyping and fluorescent in situ hybridization (FISH) [6,7]. In the 2000s, chromosomal microarray analysis (CMA) using amniocytes or CVS to provide a comprehensive genetic profile in pregnancies with suspected fetal genetic disease was recommended by ACOG and ISUOG as the first line test in high-risk pregnancies with an abnormal ultrasound [8,9]. More recently, fetal exome sequencing has been considered as a diagnostic test to identify the genetic cause of fetal genetic disorders [8]. However, the complete profiling of amniocytes or CVS using multiple technologies, in a tiered fashion or simultaneously, is time consuming and cost prohibitive. The sensitivity and specificity of karyotyping can affect reporting, as structural variant (SV) locations and sizes remain inaccurate and some translocations remain cryptic. FISH panels require *a priori* knowledge and it is difficult to detect all common microdeletions and microduplications. CMA, despite having higher resolution for the detection of copy number variants (CNVs) is limited in its ability to detect balanced translocations, inversions and low-level mosaicism [10]. Fetal exome sequencing can identify sequence variants and some copy number aberrations, but sequencing technology bias prevents the detection of several classes of large SVs [8]. 

For the past several decades, cytogenomics has been at a standstill in prenatal diagnostics and there exists a need for a disruptive, novel, and high-resolution technology that can detect clinically significant SVs in a single assay. Optical genome mapping (OGM) has been recognized as a key genomic technology for the detection of all classes of SVs in many disorders [11,12,13]. OGM has been extensively utilized to characterize SVs in postnatal and hematologic diagnostics, demonstrating a 100% clinical concordance with traditional cytogenetic analysis, and identifying additional clinically relevant abnormalities that remained beyond the purview of current technologies [11,12,14,15]. Recently, OGM performed with the Saphyr^®^ system demonstrated a clinical concordance of 100% when compared to combined cytogenetic analysis for the detection of 100 abnormalities in a cohort of 85 patients with constitutional disorders that included several sample types such as amniocytes, CVS and lymphoblastoid cells [11]. The study included 34 microdeletions or duplications, 7 aneuploidies, 28 balanced translocations as well as ring chromosomes, inversions and other complex rearrangements. This diverse set of abnormalities represents a good selection of SV classes and serves as a good foundation for exploring more difficult variations and mixtures. A study published by Shieh et al. [12] attempted to find genetic diagnoses for 50 cases of individuals with developmental delay or intellectual disability where the standard of care had failed to provide a definitive diagnosis. Within this cohort, they were able to find pathogenic or likely pathogenic variations in 12% of cases using OGM. 

Prenatal Fragile X testing may be recommended if there is a history of Fragile X in a family or if the mother is a carrier. In such cases, a specialized test is performed, sometimes in conjunction with other diagnostic tests. Supporting the ability to effectively assay for repeat expansions, Otero et al. have measured repeat expansion in DM1 and DMPK from patients with myotonic dystrophy using OGM and found repeats that ranged up to 15 kbp and they were able to show that the repeat length correlated with the degree of mRNA splicing and severity of symptoms [16]. 

Three studies of the application of OGM have recently demonstrated its ability to identify multiple genomic aberrations associated with hematologic malignancies. The first study aimed to detect all clinically relevant SVs reported by multiple methods, including karyotyping, CMA, and FISH, in leukemias from bone marrow or peripheral blood from 48 cases. The authors were able to detect all SVs and CNVs that were above 10% allele fraction in all 48 cases including several different IGH fusions, *BCR-ABL1* translocations, etc. [17]. The second study focused on acute myelogenous leukemia (AML) in 100 cases and the authors were also able to detect with OGM all clinically reported genomic abnormalities [14]. This study also demonstrated the identification of SVs deemed clinically relevant according to European Leukemia-Net (ELN) guidelines [18] in an additional 11% of cases. The third study aimed to comprehensively define SVs in myelodysplastic syndromes (MDS) [15]. In this study, the authors reported detection of all clinically actionable genomic aberrations that were above the limit of detection and found additional SVs in 33% of patients, which were later confirmed by orthogonal methods.

To date, multiple studies have demonstrated the performance of OGM and its ability to stand out as a unique technology for the detection of all classes of clinically significant genome-wide SVs. Here, we present the methodology and the application of OGM in the prenatal setting, with classical examples of several syndromic SVs that are important for the diagnosis and detection of prenatal genetic disorders. 

## 2. Prenatal OGM Workflow

**Figure 1:**
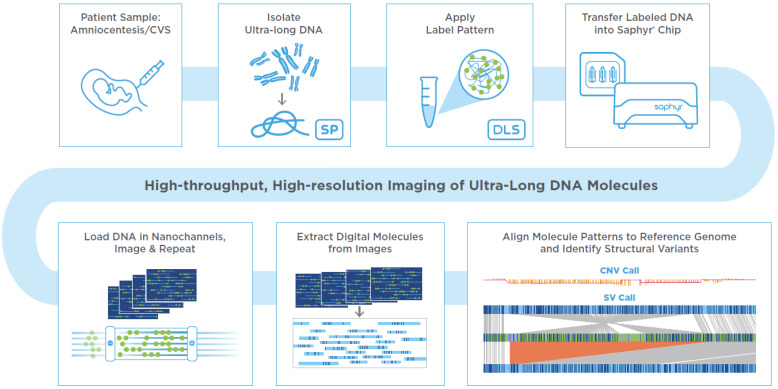
Prenatal workflow for optical genome mapping. From top left to bottom right: Sample format can be from 1 million cells, cultured or directly from cells contained in amniotic fluid or chorionic villus sampling (CVS) sample and can be fresh or frozen. DNA is subsequently labelled at a 6 bp motif by the DLS labeling technology creating a label pattern that spans the whole genome and is unique to each individual sample. Labelled DNA is then loaded on a Saphyr Chip where DNA molecules are electrophoresed into nanochannels where they are uniformly linearized for imaging by the Saphyr instrument in repeated cycles. Images are processed to extract molecules that contain the linear positions of sequence motif labels. Multiple molecules are used to create consensus genome maps representing different alleles from the sample. The sample’s unique optical genome map is aligned to the reference genome and differences are automatically called, allowing for detection of structural variations in a genome wide fashion. (Image modified from: https://bionanogenomics.com, accessed on 2 December 2020).

An assay based on OGM with the Saphyr system, a commercially available platform for genome analysis from Bionano Genomics has been developed to utilize either direct or cultured amniotic fluid cells or chorionic villus samples (CVS), which are used in clinical practice for the current standard of care methods such as karyotyping, FISH or CMA. If the amniotic fluid does not contain 1.5 million cells for proceeding with DNA extractions, it is recommended to perform cell culturing. Similarly, if the microscopic dissections of the CVS do not yield enough starting material, cell culturing is recommended. In this study, amniocyte cultures or lymphoblastoid cell lines were evaluated. First, cells were trypsinized or dislodged by gentle pipetting when they reached approximately 80–90% confluency, respectively. Cells were pelleted by centrifugation and resuspended in a cell buffer (Bionano Genomics, Inc., San Diego, CA, USA) for ultra-high molecular weight (UHMW) DNA extraction via the Bionano Prep SP DNA Isolation Kit. Subsequently, the Bionano Prep DLS Labeling Kit was used to fluorescently label long molecules at specific sequence motifs throughout the genome. The labeled DNA was loaded onto Saphyr chips for linearization and imaging in massively parallel nanochannel arrays. The observed unique patterns on single long DNA molecules were used for de novo genome assembly and structural variant calling via the Bionano Solve pipeline (version 3.6) (Figure 1). The study was approved by the IRB A- BIOMEDICAL I (IRB REGISTRATION #00000150), Augusta University. HAC IRB # 611298. Based on the IRB approval, all PHI were removed, and all data was anonymized before accessing for the study.

### 2.1. Assay Quality Control

The OGM protocol has several quality control (QC) metrics at both pre-analytical and analytical stages. The pre-analytical QC metrics include the presence of UHMW DNA—observable viscosity/clarity of DNA during pipetting and a minimum DNA concentration of >35 ng/µL needed for subsequent labeling. The analytical QC metrics include label density of ~15/100 kbp, average filtered N50 > 230 kbp, map rate > 70%, and effective coverage of >80× for the generation of a de novo assembly. All cultured amniocytes met these QC metrics, indicating that the Bionano Genomics prenatal workflow can generate high quality data similar to other already established workflows.

### 2.2. Data Analysis

Bionano Access (version 1.6), an OGM specific structural variant analysis software available as a standard web browser application, links to bioinformatic servers running Bionano Solve (version 3.6), an automated analytical pipeline for the detection of genomic abnormalities, used for data processing. Briefly, single molecules were used to generate de novo assembly of the genome. Direct alignment of maps that result from de novo assembly of single molecules and direct alignment of single molecules to the reference genome revealed SVs, CNVs as well as aneuploidies. Table 1 shows a full list of detectable variant classes via Bionano Solve. Of note, this assay based on OGM does not currently detect triploidy/tetraploidy or regions of homozygosity.

Bionano Access has numerous built-in variant filtration options that can be used to expedite the identification of pathogenic genetic aberrations. Specifically, one of the most useful features is the custom-built database of SV consisting of >300 healthy individuals that allows a fast and efficient way to filter common, likely-benign variants and generating a list of rare, potentially deleterious SVs. Additionally, users can investigate variants overlapping with specific disease-causing genes or genetic loci via a specified user generated or already available gene lists. Other useful filtration criteria include SV confidence, size and SV supporting molecule cutoffs (i.e., number of molecules confirming the identified SV). This process generally results in a small list of SVs requiring an additional review for pathogenicity classification [11,12,13,14].

### 2.3. OGM Turnaround Time

The OGM workflow using the Saphyr system is optimized to deliver fast turnaround times. UHMW DNA extraction, molecule labeling and instrument run can all be accomplished in 3 days. As in standard cytogenomic procedures in a prenatal workflow, extra time may be allocated for cell culture growth. Automated data analysis pipelines with scalable cloud computing support, web interface and variant filtrations settings allow for fast processing for raw data into an actionable report.

## 3. Clinical Significance and Representative Examples of SVs in Prenatal/Postnatal Setting

Screening for open neural tube defects and chromosomal aberrations is an important part of prenatal care. NIPT as a global screening test has changed the prenatal testing landscape dramatically in the past decade. The American College of Obstetricians and Gynecologists (ACOG) recently recommended that prenatal aneuploidy screening should be offered to all pregnant women regardless of age or known risk factors, compared to the previous recommendation where the screening for chromosomal anomalies was offered only to women >35 years, or with previous history of miscarriages [1]. 

**Figure 2:**
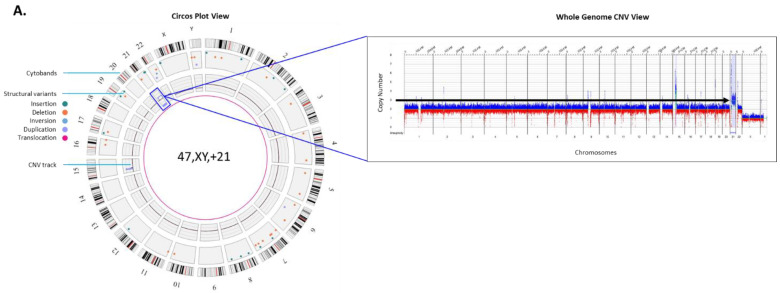

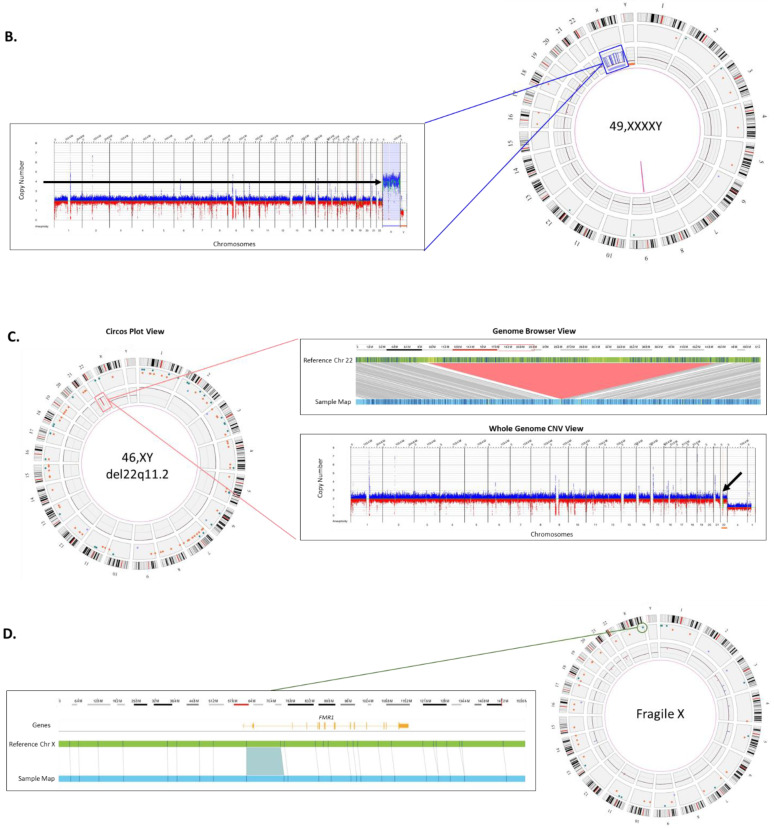

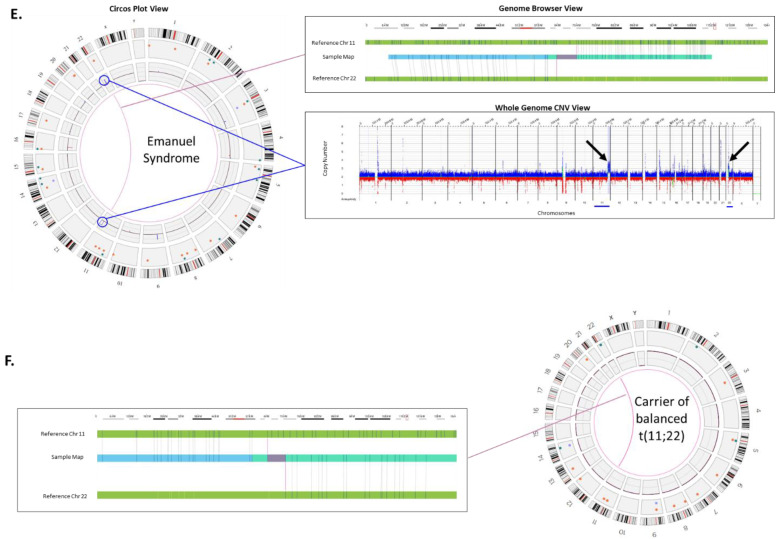
Examples of structural variants (SVs) identified by Optical genome mapping (OGM). OGM circos plots are automatically generated and the default view is composed of the following layers: the outer circle displays cytoband locations, the middle circle displays color-coded interstitial SVs that were identified in those particular locations, and the innermost circle displays observed copy number changes for each chromosome or region. Translocations are reported as lines in the center connecting the genomic loci involved. (**A**) Left panel: represents the circos plot with a copy number gain visible and highlighted with a blue box on the circos plot around the inner circle of the CNV plot highlights the chromosome 21 gain, the right panel shows the whole genome CNV profile, a linear visualization of the CNV changes across the genome. The *Y*-axis represents the copy number change and *X*-axis lists the chromosome numbers. Gains are highlighted in blue while losses are highlighted in red. Here, the black arrow points to chromosome 21 that has 3 copies. (**B**) Right panel: shows the circos plot displaying SVs and an aneuploidy in the sample. The blue box around the inner circle of the CNV plot points to chromosome X gain. Left panel: the CNV plot shows a gain of chromosome X. The black arrow points to chromosome X, which is present in four copies. (**C**) Left panel: shows the circos plot summary displaying SVs in the sample. The orange box around a region on chromosome 22 highlights a pathogenic deletion. Top right panel: The genome browser view details the alignment of the sample’s consensus map (light blue bar) with the reference consensus maps (light green bars) and provides the detail of the structural variation. Here, the sample’s map alignment to the reference maps of chromosomes 22 illustrates a large ~3 Mbp deletion (light red). Bottom right panel: CNV plot showing loss on chromosome 22 (black arrow). (**D**) Right panel: shows the circos plot summary displaying SVs in the sample. The green circle in the middle circle highlights an insertion identified on chromosome X. Left panel: the genome browser view details the alignment of the sample’s consensus map (light blue bar) with the reference chromosome X (light green bars) showing a highlighted region on the sample map that contains an insertion. The insertion is within the *FMR1* gene, inferred (and confirmed) to be a triplet repeat expansion. (**E**) Left panel: shows the circos plot summary displaying SVs in the sample. Blue lines point to regions on chromosomes 11 and 22 with CNV gains. The purple line points to a translocation also observed between chromosomes 11 and 22. Top right panel: The genome browser view detailing the alignment of the sample’s consensus map (light blue bar) with the reference chromosome 11 and 22. Here, the sample’s map aligns to two reference chromosomes indicating a translocation. Bottom right panel: CNV plot showing CNV gains on chromosomes 11 and 22 (black arrows). (**F**) The carrier mother of the case in Figure 2E showing a balanced translocation between chromosomes 11 and 22, but no CNV gains on either chromosome 11 or 22.

The global incidence of congenital disorders is ~6% (7.9 million infants) of which 50% of birth defects remain of unknown genetic cause [19]. The chromosomal aberrations that include large duplications, deletions of chromosomal segments or entire chromosomes can be determined during prenatal care. The liveborn trisomic syndromes such as, trisomy13 (Patau syndrome), 18 (Edwards syndrome) and 21 (Down syndrome) account for some of the most common birth defects [20]. Hence, NIPT has become the first tier screening test for these autosomal liveborn trisomies (trisomy 13, 18 and 21) and sex chromosomal aneuploidies (Turner syndrome, Klinefelter syndrome, etc.) [1,2,3]. Not only must these NIPT screens be confirmed with invasive diagnostic testing (on amnio and CVS samples), which is currently performed by karyotyping, FISH and CMA, but other genomic aberrations are detected by invasive testing (beyond the scope of NIPT assays as a genome-wide screen). Taken together, these technologies are time consuming, limited in resolution and cost-prohibitive. The application of OGM with Bionano’s Saphyr system to develop assays in clinical diagnostics is revolutionizing the practice of cytogenetics and is becoming a next-generation cytogenomic tool with the potential to replace the traditional cytogenetic methods in the coming years. In this commentary, we present a few examples of unique SV classes that have been identified by OGM (Figure 2, examples derived from cultured amniocytes, whole blood, or cell lines). 

**Autosomal trisomy:** Trisomy 21, or Down syndrome is the most common live-born trisomy syndrome [20]. Soft ultrasonography markers allude to the presence of trisomy 21 and liveborn babies have distinct facial and physical features, with congenital malformations including cardiovascular system, gastrointestinal tract and immune system [21]. The example in Figure 2A shows the genome data in a circos plot as well as the copy number plot showing a gain of chromosome 21 identified with the OGM prenatal workflow. 

**Sex Chromosome Aneuploidy:** Disorders of sexual development (DSD), which include sex chromosome aneuploidies, affect approximately 0.5% of the global population. The spectrum of phenotypes for cases affected with DSD ranges from mild non-syndromic forms such as hypospadias to severe syndromic forms with complete sex reversal [22]. Figure 2B shows an example of a sex-chromosome aneuploidy case identified by OGM. Four copies of the X chromosome are present in the patient as seen by the circos plot on the left and linear representation of chromosomal copy number changes on the right.

**Microdeletion syndrome:** DiGeorge syndrome is the most common microdeletion syndrome affecting approximately 1:4000 births, requiring preventive measures at birth. It occurs as a result of a ~3 Mb deletion at Chr 22q11.2 and the clinical symptoms include congenital heart defects, developmental delay, and hypocalcemia among other symptoms [23]. Figure 2C shows an example of the classical 2.87 Mb deletion of Chr 22q11.2. This deletion can be seen by the two complementary calling algorithms: the CNV profile provides dosage information and can be seen as a red dip in the circos plot (boxed) and also in the whole genome CNV display (arrow). The fusion for genomic DNA adjacent to the deletion provides further evidence of the deletion and gives high accuracy of the deletion breakpoints. 

**Repeat expansion disorders:** Certain triplet, tetra-, penta-, and hexa-nucleotide sequences can expand for thousands of base pairs resulting in pathogenic phenotypes such as Fragile X, amyotrophic lateral sclerosis (ALS), and myotonic dystrophy [24]. Accurately detecting and measuring these repeat expansions is difficult since long tandem repeat measurement is refractory to molecular methods and too small to be detected by cytogenetic methods. Figure 2D shows the repeat expansion in the *FMR1* gene causing Fragile X syndrome. The measurement of the size of the DNA fragment between two flanking labels is precise, within ~60 base-pairs, or 20 repeat units. Therefore, an expansion above ~>220 repeats could be utilized to “rule-in” a pathogenic finding. In the example shown, the expansion is 867 bp or 289 triplet repeats. OGM can effectively size repeat expansions that are at least 500 bp and up to 100 kbp in size.

**Unbalanced translocations (in fetus) secondary to a balanced translocation carrier (parent):** Emanuel Syndrome is caused by a chromosomal imbalance secondary to a parental balanced translocation involving chromosomes 11 and 22. The unbalanced +der (22) results are due to the malsegregation of one derivative chromosome resulting in 47 chromosomes. The extra chromosome leaves the affected individual with three copies of portions of chromosomes 11 and 22 [25]. Figure 2E shows the OGM result for an individual with Emanuel syndrome, in this case the copy increases can be seen on chromosome 11 and 22 in the circos plot (circled) and in the whole genome CNV plot (arrows). Furthermore, the fusion of chromosome 11 and 22 can be seen in the circos plot by the pink line connecting the two chromosomes in the center. An expanded view of the fusion in the genome browser shows where a genome map from the case can be seen connecting the two chromosomes. The most frequent balanced translocation in the human population, t(11;22) [25], was detected in the carrier parent as highlighted in Figure 2F. OGM was also able to detect the carrier status through direct detection of the inter-chromosomal fusion in copy number neutral cells (Figure 2F). 

To demonstrate the inter-site reproducibility of the OGM prenatal workflow, the results from five cultured amniocytes samples were replicated at two different sites, by different instruments and different operators (unpublished data), indicating the robustness of the assay based on this method. In this reproducibility study, all assays passed the QC metrics at both sites and all clinical aberrations were detected at both sites. Larger reproducibility studies will be needed in the future to further establish the robustness. Nevertheless, since data processing and SV calling by OGM with the Saphyr system is much more automated compared to traditional cytogenetics, it is more objective and therefore expected to have fewer subjective operator-determined differences (as commonly observed with skilled cytogenetic personnel in any clinical laboratory). This simplicity and objectivity in data interpretation also significantly reduces the burden of highly trained experts. 

This short communication demonstrates the power and potential of OGM in detecting the classical genomic abnormalities that are commonly found in prenatal testing. Combining structural variations, copy number variations and repeat expansions into a single assay is unprecedented for any genomic technology. 

## 4. Conclusions

Optical genome mapping, with its power to detect all classes of SVs, including CNVs, at a higher resolution than traditional cytogenetic methods can play a significant role in prenatal care and management as a next-generation cytogenomic tool. OGM can be employed as an invasive prenatal testing tool after a positive NIPT screen and for the detection of microdeletion/duplication syndromes implicated in high-risk pregnancies. OGM has shown 100% concordance with the current combinatorial cytogenetic assays in several studies aimed at investigating complex genetic disorders, constitutional disorders and liquid tumors, while detecting all of the different types of chromosomal anomalies including aneuploidies, large deletions/duplications, CNVs, balanced chromosomal events and complex chromosomal rearrangements. Currently, in clinical practice, small (<25 kb) structural and copy number variations are not reported, unless gene specific analysis is performed to detect intragenic CNVs. However, OGM demonstrates the ability to detect large reportable SVs/CNVs as well as small SVs accurately, e.g., Fragile X expansions (not detected by routine cytogenetic methods but an important application in both prenatal and postnatal settings). The detection of the entire spectrum of cytogenetic aberration in a single assay, with the capability of identifying unique genomic abnormalities and the ability to better characterize the SVs, demonstrates the potential of OGM technology to be used as a single alternative to traditional cytogenetic assays. Additionally, as several publications have shown, OGM has the potential to identify novel, clinically significant genetic abnormalities, which can increase the diagnostic yield not only in the clinic, but also in the prenatal setting. 

To conclude, assays based on OGM are cost-effective, have fast turn-around times with sample to reporting in 4 days, do not require custom complex bioinformatic pipelines, are high-resolution compared to traditional methods and have the ability to detect all SV classes relevant for pre-natal diagnostic testing in an automated fashion. 

## Figures and Tables

**Table 1:** Specification of optical genome mapping for the detection of different variant classes.

Variant	Variant Types		Variant Description	Bionano OGM
Aneuploidy	Monosomy		Chromosome loss	✓
	Trisomy		Chromosome gain	✓
	Triploidy		Whole genome Triploidy	Not currently
	Tetraploidy		Whole genome Tetraploidy	Not currently
	Ring chromosome		CNV and fusion	≥500 kbp + fusion break
Structural Variants	Copy Number Variants	Deletions/Duplications	Interstitial	≥500 bp
			Terminal	≥500 kbp
		Insertions	Interstitial (unknown sequence)	≥500 bp
	Translocations		Balanced	✓
			Unbalanced	✓
	Inversions		Pericentric	✓
			Paracentric	≥30 kbp
Regions of Homozygosity	ROH		ROH	In development
Macrosatellite/microsatellite	Repeats		Contractions/expansions	≥500 bp
Sequencing Variants	Single nucleotide variants, INDELs		Transitions/transversions Insertions/deletions <50 bp	–

## Data Availability

All relevant data has been included in the manuscript.
